# National Sanatorium for Workers Suffering from Tuberculosis, Benenden, Kent

**Published:** 1907-05-04

**Authors:** 


### NATIONAL SANATORIUM FOR WORKERS SUFFERING FROM TUBERCULOSIS, BENENDEN, KENT.

On April 30 H.R.H. Princess Christian, who was accompanied by Princess Victoria of Schleswig-Holstein, went to Benenden to inspect and formally inaugurate the completed portion of the sanatorium for sufferers from tuberculosis which is being built there. Her Royal Highness was received by Sir Clifton Robinson, and Lady Robinson presented her with a bouquet of lilac and orchids, while Princess Victoria accepted one of pink roses and white orchids. A special train conveyed a number of those interested in the institution to Cranbrook, whence they journeyed by motor to Benenden. The sanatorium is situated on rising ground about six miles from Cranbrook station, and overlooks a wide expanse of rolling country. It is a two-storey building, shaped like a crescent, and facing south. There are twenty single rooms and twenty-eight double-bedded ones, and as the depth of the building is only that of the room and the corridor behind it, through ventilation is easily maintained. The rooms are warmed by closed anthracite stoves. The method of building is simple and economical. No money has been wasted on mere architectural ornament, although the institution has a pleasant and attractive aspect. The construction is of steel framing, the space between the stanchions and girders being filled in with Frazzi cement, rough-casted on the outside and plastered on the inside. It is estimated that the cost of the building when completed will not be more than £100 per bed. Already there are nineteen male patients in residence, under the care of the matron, Miss S. A. Musson, a sister and two nurses. The site of the sanatorium includes 250 acres of farmland, and it is hoped that such patients as are able will learn there the elements of agricultural work, and thus be induced to take up such employments on their restoration to health as will keep them in rural surroundings. In the course of a speech to those assembled, Sir Clifton Robinson pointed out that a few years ago the idea prevailed that the one great remedy for consumption and kindred complaints was a voyage to Australia. Such a policy represented mainly a counsel of despair. As a rule it was adopted only as a last resource. The length and cost of the journey, the breaking up of home ties, the severance of lifelong associations, and the doubt and uncertainty with which the future was surrounded, were considerations that generally led to the postponement of the journey—when it could be made at all—until the last possible moment. By that time the disease had probably strengthened its hold upon the sufferer, and even if her survived for any length of time in the Colonies, he was often little fitted to begin there the battle of life all over again. The sanatorium treatment was practically that of the Australian voyage—namely, the inhaling of an abundance of fresh air—and could be as well obtained in our own country, while it has the further advantages that the treatment can be begun at a much earlier stage, and therefore the cure rendered more probable; that there is not the same severance of home ties; that the sufferer can sooner become a useful member of society; and that this remedy is available for persons who could not have afforded the old. Sir Clifton hoped that the sanatorium movement would also lead to some supplementary organisation by means of which the workers who were brought out into the country and cured more or less effectually by the treatment would be enabled to spend the rest of their days in fresh air, instead of having to return to their squalid surroundings in the densest centres of overcrowded towns.

